# Supplementary material for: A light carbon isotope composition for the Sun
Source: Nat Commun. 2018 Mar 2;9:908. doi: 10.1038/s41467-018-03093-3 (PMC5834507; doi:10.1038/s41467-018-03093-3)
Supplement: Supplementary file 1 — Supplementary Information [file 41467_2018_3093_MOESM1_ESM.pdf]

Supplementary Information

Supplementary Table 1. Inefficient Coulomb drag parameters for several charge states of O and C ions

	Q = +4	Q = +5	Q = +6	Q = +7
$H_{^{16}O}$		1.0720	0.7158	0.5049
$H_{^{17}O}$		1.1525	0.7717	0.5460
$H_{^{18}O}$		1.2329	0.8276	0.5871
$f_{16,18}$		1.1110	1.0604	1.0392
$f_{16,17}$		1.0527	1.0293	1.0193
$H_{^{12}C}$	1.2360	0.7494	0.4915	
$H_{^{13}C}$	1.3620	0.8302	0.5477	
$f_{12,13}$	1.0956	1.0437	1.0263	
